# The Physiology of Sleep

**Published:** 1879-01

**Authors:** E. P. Hurd

**Affiliations:** Newburyport


### THE PHYSIOLOGY OF SLEEP.

By E. P. HURD, M.D., Newburyport.

It is well to be cautious how we accept theories, for one stubborn fact, as Tyndall has said, will upset a whole freight train of theories. It once appeared to us settled that the proximate cause of sleep was anæmia of the cortical gray matter,—the centres of ideation, comparison, and volition. The theory worked very well. A *priori* it appeared quite probable that less blood should go to the brain during its quiescence than during wakeful activity.

Moreover, there were not wanting observations to suggest and confirm this view. During intense thought and passion did we not see the temporals swell and throb and the face become turgid, and during the insensibility of syncope, epilepsy, catalepsy, did not the external appearances favor the opinion that the brain was relatively anæmic ? Must there not be relative anæmia during the insensibility of sleep also ?

We had carefully read and noted Monroe's observations and conclusions as to the contracted condition of the arterioles and the pallor of the convolutions during sleep; these had been confirmed by Hammond and Brown-Sequard. Durham's investigations on the physiology of sleep had been published as early as 1860, and seemed to be exceptionally careful and accurate. Pierquin a little earlier recorded an interesting series of observations on a girl in Montpellier who had lost a large portion of her scalp and skull. "Her brain could be seen for a considerable extent of surface. When she was in dreamless sleep her brain was motionless and low within the cranium; but when her sleep was imperfect and she was agitated with dreams, her brain moved and beat, more blood was sent to it, the arteries were relaxed, and the brain protruded through the opening in the skull. When she was awake the same difference was observed, in accordance with the activity or quiescence of her mind."

Donders "made a cruel but striking experiment. He cut away a part of the skull of an animal, and cemented in its place a piece of glass, and observed that in the waking state the brain is larger than it is during sleep, while in the latter condition it is pale and bloodless." Brown-Sequard, in his Lecons sur les Nerfs vaso-moteurs, had given his assent, based on similar observations, to the same view, and had said that sleep "resembled a light attack of epilepsy." If any further support to the *anœmia* theory was needed, it seemed to be given and all the demands of rigorous criticism satisfied when, in 1869, Hammond, of New York, published his little work on Sleep and its Derangements, wherein a number of independent researches pointed to the same conclusion.

It was very convenient to have a good theory of causation, for now it was plain how hypnotics must act, and what were the indications in insomnia. If chloral, bromides, anæsthetic and narcotic agents caused sleep, it would seem to be, for the most part, by contracting the cerebral blood-vessels, and thus diminishing the blood supply. The vaso-motor system had the principal part in this physiological change. To be sure, experimental researches had given contradictory results, but on the whole there seemed to be a considerable number of facts that went to show that hypnotics did so act.

Alas for human liability to err! We thought that all this was settled, but we were mistaken, and new experiments and new observations are imperatively needed, if, indeed, anything more concerning the intra-cranial circulation and other physiological changes during sleep and allied states of unconsciousness can ever be determined.

M. Vulpian's thorough and seemingly exhaustive researches on the physiology and pathology of the vaso-motor system have been fruitful in important results. Called in 1873 to the chair of experimental and comparative physiology, rendered vacant by the resignation of Brown-Sequard, he delivered a brilliant course of lectures on the vaso-motor system, which have been recently published in two volumes. Whatever merit may be attached to his former work, published in 1866, Lecons sur la Pkysiologie generale et comparee du Systeme neeveux—a work which has deservedly become classical—is, we think, eclipsed by this recent work of M. Vulpian.

After investigating the *role* of the sympathetic in various functional nervous diseases, where too important a part seems to have been assigned to the vaso-motors of the brain and spinal cord—*reflex paralysis*, the mechanism of whose production, he thinks, is altogether different from that taught by Brown-Sequard; *hysteria*, the motility of whose phenomena is the expression of real modifications of the elements of the cortical substance of the nerve centres, not appreciable to gross or even microscopic vision, and easily reparable; *tetanus*, *hydrophobia*, where, whatever the morbid erethism of the bulbo-spinal centres, there is no constant vascular lesion, and where, to account for the frightful manifestations, we must again resort to the vague term "molecular modifications," inappreciable, it is true, but real; *epilepsy*, where, again, Brown-Sequard's elaborate theories of vaso-motor action are found inadequate to explain the principal phenomena of the attack, —M. Vulpian devotes part of a long chapter (Dix-Huitieme Lecon) to the consideration of the physiology of sleep and the theory of cerebral anæmia. The question which he proposes for discussion is the following: "Whether the minute vessels of the encephalon undergo constant modifications in size and in contents in correspondence with the activity or want of activity of the brain; if the blood-vessels are relatively full when the hemispheres are working, and relatively empty when these centres are in a state of repose." There are two ways of reaching a solution of this question: one is by observations of the encephalon during natural sleep; the other by experiments made with hypnotic agents, and observing the results, a portion of the skull of an animal having previously been removed. M. Vulpian has performed numerous experiments which go to show that the profession has been too hasty in adopting the conclusions of Durham, Hammond and others, pursuing a similar line of investigation. It is quite true that the observations of these experimental physiologists were correct; the error was in concluding that, because in certain instances the arterioles were found contracted and the brain pale and sunken during sleep, therefore the anæmic condition was the procuring cause of sleep. Vulpian admits that in ordinary sleep less blood circulates in the cerebral centres than when awake, but this relative anæmia is rather the concomitant, or result, than cause of the suspension of cerebral activity. "I am compelled to believe that during the first moments of sleep there is a degree, more or less marked, of encephalic congestion analogous to the congestion of the face and of the conjunctivæ which is observed at the same time. But in all probability, the respiration soon becoming calmer, more regular, a little less frequent, the movements of the heart becoming a little slower and less energetic, the cerebral circulation must undergo a like modification, and the congestion at the onset will give place to a slight degree of relative anæmia. Perhaps, also, the vaso-motor apparatus, by reason of the relative functional repose of the cerebrum, takes on a slight predominance of action, having for conseqence a state of feeble augmentation of the *tonus* of the different vessels of the organism, those of the encephalon included."

Among the objections to the *anœmia theory* is this one, forcibly stated by Vulpian: that in individuals suffering from *anœmia*, of whatever cause (hæmorrhages, chlorosis, cachexia), there is ordinarily a marked tendency to insomnia. If the theory of Durham were well founded, one ought, in these conditions, to find a disposition to somnolence. On the other hand, in states of plethora the tendency to sleep is often very conspicuous. It is so in general after a full meal. To talk about the replete blood-vessels of the stomach and intestines, after a repast, *driving* blood from the encephalon, seems nonsense when we remark the turgid countenance, the injected eyes, the throbbing temporals, of the sleeping gourmand.

Another objection to the theory is the fact that the results of ligation of the encephalic blood-vessels do not favor it. Vulpian, after numerous trials in this direction, declares that "it is impossible, in practicing graduated compression of the encephalic vessels, to produce sleep at any moment whatever of the experiment." Moreover in animals, faradization of the cephalic ends of the two cervical sympathetic nerves has repeatedly been practiced. "The result was some amount of cerebral anæmia [*un certain degre, assez faible relativement*] without the least manifestation of somnolence."

It would seem that if there were any causal connection between excitation of the vaso-constrictor nerves of the encephalon and sleep, electrization of the sympathetic in the neck should cause somnolence; nothing like somnolence occurred in Vulpian's experiments.

How about the action of anæsthetics and hypnotics? Hammond's experiments with ether determined a decided pallor of the surface of the brain in animals on which he operated. Vulpian's experiments, many of which were made before his class, were not attended with any marked or constant modification of the blood-vessels in the brain, whether the inspection were made with the naked eye or with a lens.

Other experiments, made with chloroform, equally failed to show any marked effect on the blood-vessels; "the cerebral anæmia, inconspicuous under etherization, is still less marked during the insensibility of chloroform." As for *opium*, its effects on the little vessels of the pia mater appeared to be *nil.* The opium in solution was injected under the skin; in half an hour the animal was in a state of profound somnolence; "the vascularity of the cerebrum was not changed in any appreciable manner."

Other experiments were made with hydrate of chloral. Deep, sleep resulted; the encephalon, denuded of a portion of its cranial covering, was inspected; no modification in the condition of the vessels of the pia mater were discernible.

Vulpian's conclusions from his carefully conducted experiments is that sleep natural and artificial, is a phenomenon essentially independent of the vaso-motor system and the state of the blood-vessels. The anatomical elements of the gray substance of certain parts of the encephalon have a *habit,* at certain times, and under certain conditions, of *lapsing into a*

*state of functional inactivity.* It is an "*engourdissement,*"—a torpor. The vascular, cardiac, and other manifestations are only accessory; they may be concomitant or consecutive, but they do not play any essential *role* in the physiology of sleep.

It seems to me probable that this may be for some time to come the last word on the subject, and that neither *this* condition of the blood-vessels nor *that* condition is the physiological antecedent of sleep; that, in fact, we must seek for the cause of this periodical phenomenon, so essential to our well-being, so suggestive, moreover, of the essential *oneness* of the physical and psychological—body and mind—in that invisible world of molecules whose polarities, permutations, and combinations produce results that baffle intellect and imagination to comprehend, and make nature herself transcendental.

It is evident that for a long time to come hypnotic agents must be given from empirical considerations solely, and not from any supposed knowledge of their *modus operandi.* Of course, it will be quite proper to indulge in any amount of reasonable speculation as to how thec erebral cells are affected by drugs, and it must be admitted that considering the unfathomable mysteries of the molecular world, of which we get some hints, we must not be surprised at any future revelation concerning them, just as Lord Beaconsfield says that we need not be surprised at anything which takes place under a republican form of government!

It is doubtless true that while chloral and soporifics generally do not act by shutting off in part the arterial blood current, yet since a certain amount of relative anæmia is generally an accompaniment of natural sleep,—a full supply of oxygenated blood being essential to active cerebration,—and since the *minimum* of cerebration (with arrest of those destructive changes which are the correlatives of states of conciousness) is hardly compatible with the *maximum* of arterial blood supply, measures tending to diminish the amount of blood circulating in the brain, such as cold to the head, and derivatives, are indicated in insomnia. These will often fail where, were the anæmia theory true, they should succeed. The causes of insomnia are very generally subjective, and no remedy is effective which does not include a radical change in the habits, modes of thought and feeling, and surroundings of the patient.

